# CNS Border-Associated Macrophages: Ontogeny and Potential Implication in Disease

**DOI:** 10.3390/cimb45050272

**Published:** 2023-05-13

**Authors:** Iasonas Dermitzakis, Paschalis Theotokis, Paschalis Evangelidis, Efthymia Delilampou, Nikolaos Evangelidis, Anastasia Chatzisavvidou, Eleni Avramidou, Maria Eleni Manthou

**Affiliations:** Department of Histology-Embryology, School of Medicine, Aristotle University of Thessaloniki, 54124 Thessaloniki, Greece; iasonasd@auth.gr (I.D.); ptheotokis@auth.gr (P.T.); pascevan@auth.gr (P.E.); edelilamp@auth.gr (E.D.); evangeln@auth.gr (N.E.); achatzisav@auth.gr (A.C.); avramidoue@auth.gr (E.A.)

**Keywords:** CNS border-associated macrophages, tissue-resident macrophages, origin, yolk sac, molecular cues, development, disease

## Abstract

Being immune privileged, the central nervous system (CNS) is constituted by unique parenchymal and non-parenchymal tissue-resident macrophages, namely, microglia and border-associated macrophages (BAMs), respectively. BAMs are found in the choroid plexus, meningeal and perivascular spaces, playing critical roles in maintaining CNS homeostasis while being phenotypically and functionally distinct from microglial cells. Although the ontogeny of microglia has been largely determined, BAMs need comparable scrutiny as they have been recently discovered and have not been thoroughly explored. Newly developed techniques have transformed our understanding of BAMs, revealing their cellular heterogeneity and diversity. Recent data showed that BAMs also originate from yolk sac progenitors instead of bone marrow-derived monocytes, highlighting the absolute need to further investigate their repopulation pattern in adult CNS. Shedding light on the molecular cues and drivers orchestrating BAM generation is essential for delineating their cellular identity. BAMs are receiving more attention since they are gradually incorporated into neurodegenerative and neuroinflammatory disease evaluations. The present review provides insights towards the current understanding regarding the ontogeny of BAMs and their involvement in CNS diseases, paving their way into targeted therapeutic strategies and precision medicine.

## 1. Introduction

The central nervous system (CNS) is considered immune-privileged as the blood–brain barrier (BBB) prevents its overexposure to external pathogens [1]. The CNS homeostasis is also maintained by immune cells, such as tissue-resident macrophages neutralizing noxious factors that are potentially harmful due to neurons’ limited capacity for renewal [2,3]. For a long time, microglia were referred to as the main and only innate immune cells of the CNS [4]. Recently, another population of tissue-resident macrophages was distinguished from the parenchymal macrophages of the CNS [5]. These cells are called CNS border-associated macrophages (BAMs) or CNS-associated macrophages (CAMs), first described as perivascular phagocytes in rats, and are located in the non-parenchymal region of CNS [6,7]. Specifically, the meninges, choroid plexus and perivascular spaces of the brain are populated by BAMs [8].

The single-cell analysis and fate-mapping in transgenic animal models facilitate the detection of microglia and BAMs, shedding light on the cells’ molecular identity without the need for irradiation in the experimental protocols [5,9,10,11,12,13]. Indeed, BAMs were clustered in different groups depending on their anatomical positions, revealing each subset’s heterogeneity [14,15]. Specifically, ΒAMs are divided into subdural/leptomeningeal macrophages (sdΜΦ), dural macrophages (dmΜΦ), stromal choroid plexus macrophages (cpΜΦ), choroid epiplexus macrophages (cp^epi^ΜΦ), also known as Kolmer’s cells, and perivascular macrophages (pvΜΦ) [14]. The subpopulations of BAMs generally vary in morphology, motility, and function. Although BAMs display differences in their transcriptional profiles and dynamics, their functional diversity in each anatomical location has yet to be fully elucidated [16].

The overview of the origin of CNS macrophages has radically evolved during the last decades. In the past, all tissue-specific macrophages were considered to be derived from bone marrow progenitors [17,18,19]. However, according to recent data from studies that have utilized new genetic tools, the early perception that tissue-resident macrophages derive solely from adult blood circulating monocytes is no longer prevalent [8,11,20,21,22,23,24]. Although several studies have investigated the role of microglia in neuroinflammation and neurodegenerative diseases, such as Alzheimer’s disease, Parkinson’s disease and multiple sclerosis, little is known about the contribution of BAMs in these pathophysiological patterns [25,26,27,28,29,30]. There are apparent limitations to elucidating the contribution of BAMs in neurological disease; the exact pathophysiological role of BAMs and other CNS-innate immune cells cannot be interpreted by the majority of available tools; therefore, the distinction of BAMs from microglia under disease remains challenging [12,31]. Nevertheless, data obtained via contemporary technologies indicate their potential involvement and miscellaneous phenotypes in neurodegenerative and neuroinflammatory CNS diseases [5,12,32,33,34,35,36].

This review primarily aims to present the origin of BAMs in the fetal and postnatal period, the molecular cues which drive BAM generation, the principal distinction from microglia, and the implication of BAMs in various CNS disease conditions.

## 2. Origin of BAMs during Embryogenesis and Adulthood

The BAM embryonic origin was first investigated in rodents using bone marrow chimeras and whole-body irradiation, proposing that BAMs are bone marrow-derived [37,38]. In 2016, Goldmann et al., performing fate mapping analysis, observed that BAMs originate from the mouse yolk sac’s early erythro-myeloid progenitors (EMPs) during embryogenesis [8]. A tamoxifen-inducible Runx1^CreER^R26^YFP^fate-mapping mouse model confirmed that BAMs originate from early EMPs in the yolk sac, which gave rise to two different macrophage populations, namely, CD206^+^ (BAM progenitors) and CD206^−^ (microglial progenitors) without the contribution of fetal liver or definitive hematopoiesis [20]. Interestingly, the mannose receptor C-type 1 (MRC1 or CD206) is a unique marker for BAMs [8,12,14]. The expression of *Mrc1* is upregulated from E8.5 when the primitive macrophages, originating from EMPs, prepare to invade the embryonic tissues [39]. Recently, Masuda et al. investigated the progenitors of BAMs utilizing single-cell RNA sequencing and fate mapping analysis in the *Mrc1^CreERT2^* mouse model. Although flow cytometry confirmed the presence of a CD206^+^ subpopulation within the A2 cells (CD45^+^ c-kit^−^ CX_3_CR1^+^ cells), meningeal macrophages and microglia were found to originate from common CD206^+^ A2 progenitors in contrast with previous results [20,40]. The pvΜΦ were generated postnatally from sdΜΦ, requiring integrin-signaling and vascular smooth muscle cells (VSMCs) [40].

Regarding the repopulation pattern of BAMs in adulthood, there is a great heterogeneity between BAM clusters; specifically, the sdΜΦ, pvMΦ and cp^epi^ΜΦ exhibit similar longevity with microglial cells as being self-maintained in the CNS independently from blood monocytes’ contribution [8,14]. The cp^epi^ΜΦ were solely derived from local SALL1^+^ macrophages [14]. In *Ccr2*-deficient mice, the number of cpMΦ decreased, revealing their replenishment from Ly6Chi monocytes and shorter turnover [8]. In accordance with these results, Van Hove et al., combining single-cell RNA sequencing with complementary approaches in mice, suggested that dmΜΦ and cpΜΦ were gradually replenished by bone marrow-derived monocytes [14]. As dura mater and choroid plexus stroma are more accessible brain regions than (i) subdural space, (ii) the apical surface of the choroid plexuses, and (iii) brain parenchyma, the tissue permeability may be considered a crucial factor for brain macrophage ontogeny. However, the ablation of BAMs through CSF1R blockade led to the replenishment of cpΜΦ and dmΜΦ via local expansion, indicating their self-renewal capacity, while sdΜΦ presented difficulties in their repopulation [14].

By utilizing the Cx3cr1^CreER^:R26^tdTomato^ fate mapping system in an experimental autoimmune encephalomyelitis (EAE) mouse model, Jordão et al. proposed that BAMs remained stable and locally self-renewed in addition to the recruitment of bone marrow-derived progenitors [12]. In Cx3cr1^gfp^Ccr2^rfp^ bone marrow chimeric mice, CD169^+^ BAMs proliferated after ischemia, while a small proportion of BAMs was bone marrow-derived, populating the perivascular and ischemic regions [36]. Both in homeostasis and disease, skull and vertebrae bone marrow constitute a pool of myeloid cells that can invade non-parenchymal and parenchymal CNS regions, transforming into tissue-resident macrophages [41]. A fate-mapping analysis in a mouse model of Alzheimer’s disease (AD) revealed that BAMs are a stable cell population with an unaffected turnover rate and a minimal replenishment from bone marrow-derived cells during this neurodegenerative disease [42].

Summarizing, the origin of BAMs has been extensively studied in the last few years using new genetic tools, e.g., fate mapping analysis. It has been proposed that BAMs originate from early EMPs in the yolk sac during embryogenesis. Although specific BAMs are replenished by peripherally-derived monocytes postnatally, some remain solely derived from the local pool. BAMs have been shown to remain stable and locally self-renewed in both homeostasis and disease. Further investigation is needed to (i) confirm BAM origin, (ii) detect the precise embryonic progenitors of BAMs, especially of the dura mater and choroid plexus macrophages, (iii) determine the timing of each BAM subpopulation’s generation, and (iv) delineate their repopulation pattern.

## 3. Molecular Drives Orchestrating BAM Development

The transcription factor PU.1 (or SFPI) could be essential for the BAM generation during embryonic development since research has showed that in mice with deletion of the *Sfpi1* gene, pvMΦ, sdMΦ, and cpMΦ were ablated [8]. Progenitors of BAMs express the runt-related transcription factor 1 (RUNX1), which regulates the expression of PU.1 during embryogenesis [20,43]. The impairment of PU.1 factor in mice results in a reduced number of A1 (CD45^+^ c-kit^lo^ CX_3_CR1^−^ immature cells) and A2 (CD45^+^ c-kit^−^ CX_3_CR1^+^ cells) progenitor cells of the yolk sac, from which both microglial cells and BAMs originate. In contrast, the lack of interferon regulatory factor 8 (IRF8) exclusively decreased the number of A2 cells [44]. Furthermore, the colony-stimulating factor 1 receptor (CSF1R) signaling could be essential for BAM development [5,11,14]. In a zebrafish model carrying the panther mutation, a loss-of-function mutation in the *fms* gene orthologue which encodes CSF1R, primitive macrophages of the yolk sac could not colonize the embryonic tissues [45].

**Figure 1:**
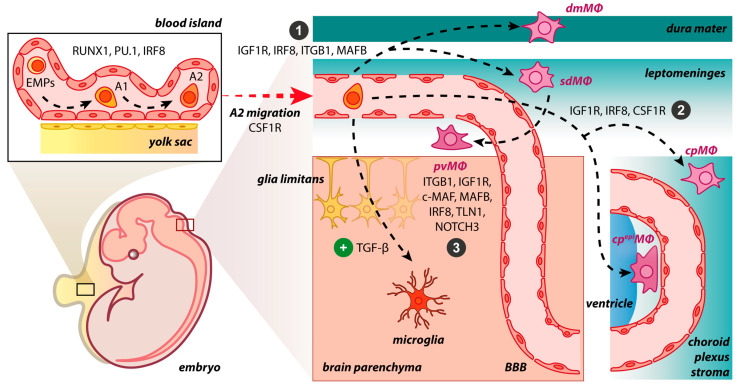
BAM origin and propagation in the developing mouse brain. The early differentiation of macrophage progenitors is regulated by the expression of RUNX1, PU.1, and IRF8 in the yolk sac, where the primitive erythro-myeloid progenitors (EMPs) give rise to CD45^+^ c-kit^lo^ CX_3_CR1^−^ immature (A1) cells and subsequently to CD45^+^ c-kit^−^ CX_3_CR1^+^ (A2) cells. In the presence of TGF-β, A2 cells initiate a microgliogenesis program upon settlement in the brain parenchyma. In the absence of the TGF-β, A2 cells do not enter the brain parenchyma, populate the abutting connective tissue, and may follow distinct developmental pathways: (1) IGF1R, IRF8, ITGB1, and MAFB restrict progenitors to the meninges, either dura or the subdural mesenchymal niche; (2) IGF1R, IRF8, and CSF1R dictate progenitors’ residency within the choroid plexus; (3) ITGB1, IGF1R, c-MAF, MAFB, IRF8, TLN1, and NOTCH3 stimulate pvΜΦ generation postnatally from sdΜΦ. However, it is not yet fully understood if the dmΜΦ share common progenitors and drivers with sdΜΦ during embryogenesis. dmΜΦ: dural macrophages; sdΜΦ: subdural macrophages; pvΜΦ: perivascular macrophages; cpΜΦ: stromal choroid plexus macrophages; cp^epi^ΜΦ: choroid epiplexus macrophages; BBB: blood–brain barrier.

After progenitors’ migration and invasion in the CNS, BAM generation is initiated (Figure 1). The BAMs may be developed independently of transforming growth factor beta receptor (TGF-βR) signaling. In *Tgfbr2*-deficient mice, no alteration in cell numbers of BAMs occurred, while transforming growth factor beta (TGF-β) is required for the generation of microglial cells [20,46]. Three main brain border regions are filled with BAMs, namely, meninges, choroid plexus, and perivascular spaces. The postnatal expansion of sdMΦ was influenced by IRF8 and MAFB [40]. Indeed, in *Irf8*-deficient mice, a reduction of sdMΦ was observed [8]. The lack of integrin subunit beta 1 (ITGB1) in mice resulted only in a minor change in the numbers of sdΜΦ [40]. Similarly, the absence of insulin-like growth factor 1 (IGF1R) induces transcriptomic changes in BAMs via its implication in RNA processing, growth, migration and intracellular signaling [47]. The MYB, BATF3, and NR4A1 transcription factors were not necessary for BAM development [8].

Specific molecular cues may also regulate the development of choroid plexus macrophages. Colony stimulating factor 1 (CSF1 or M-CSF), produced by stromal and epithelial cells, is crucial for macrophages’ ontogeny, orchestrating their proliferation and differentiation [48]. CSF1 binds to its receptor, namely, CSF1R, a homodimeric type III receptor tyrosine kinase [49]. Fms-intronic regulatory element (FIRE) is a highly conserved enhancer found in the second intron of the *Csf1r* gene [50]. In mutant mice with deletion of FIRE, the production and maintenance of cpMΦ were partially impaired [51]. On the contrary, Rojo et al. demonstrated that in FIRE-deficient mice, microglial cells were absent from the brain, whereas BAMs were retained [52]. Interestingly, cpMΦ remained unaltered in a study with *Irf8*-deficient mice [8], while other research considered IRF8 as a regulator of cpMΦ maturation since the gene ablation suppressed the transcriptional programme of cpMΦ [14,53].

The transcription factor c-MAF, a member of Maf family transcription factors, could be crucial for regulating the pvMΦ transcriptional programme as the deletion of *c-Maf* in macrophage lineages resulted in the ablation of pvMΦ in the mouse brain [54]. The postnatal expansion of pvΜΦ was also influenced by IRF8 and MAFB [40]. Moreover, VSMCs have a potential role in the distribution of the pvMΦ during development. In *Notch3*-deficient *mice*, VSMCs are reduced similarly to the pvMΦ, while the number of sdΜΦ was maintained [40,55]. The distribution of pvMΦ is also controlled by integrin signaling. *Talin 1* (*Tln1*) is an integrin-related gene which encodes a cytoskeletal protein. In *Tln1*^−/−^ mice, a significantly lower number of pvMΦ was observed, while microglia and sdΜΦ were not affected in the developing brain, underscoring the impaired vascularization as the cause of pvMΦ reduction [40]. Nevertheless, the absence of integrin subunit beta 1 (ITGB1) in mice resulted only in a minor change in the numbers of pvΜΦ [40]. All the aforementioned molecular cues involved in BAM development are presented in Table 1.

To recapitulate, the emergence of BAMs is considered a complex process tightly regulated by multiple molecular cues in a similar pattern to oligodendrogenesis and microgliogenesis [24,56]. Although some molecular drivers orchestrating BAM generation have been recently discovered, it remains a largely uncharted territory.

**Table 1:** Molecular drivers and cues regulating the development of BAMs.

Gene	Locus	Protein	Location	Tissue Specificity	Brain Specificity	Molecular Function	Species	Ref.
*CSF1R*	5q32	Colony stimulating factor 1 receptor	Vesicles; Plasma membrane	Lymphoid tissue; Placenta	Low	Kinase; Receptor; Transferase	Mice; Zebrafish	[5,11,14,45,51,52]
*IGF1R*	15q26.3	Insulin-like growth factor 1 receptor	Plasma membrane	Low	Low	Kinase; Receptor; Transferase	Mice	[47]
*IRF8*	16q24.1	Interferon regulatory factor 8	Nucleoplasm	Bone marrow; Lymphoid tissue	Low	Activator; DNA-binding; Repressor	Mice	[8,14,40,44,53]
*ITGB1*	10p11.22	Integrin subunit beta 1	Plasma membrane; Focal adhesion sites; Endoplasmic reticulum	Low	Low	Virus entry; Integrin; Receptor	Mice	[40]
*MAF*	16q23.2	MAF bZIP transcription factor	Nucleoplasm; Nuclear bodies; Vesicles	Low	Low	Activator; DNA-binding; Repressor	Mice	[54]
*MAFB*	20q12	MAF bZIP transcription factor B	Nucleoplasm; Nucleoli; Golgi apparatus; Cytosol	Parathyroid gland	Low	Activator; DNA-binding; Repressor	Mice	[40]
*NOTCH3*	19p13.12	Notch receptor 3	Nucleoplasm; Cytosol; Actin filaments	Low	Low	Activator; Developmental protein; Receptor	Mice	[40,55]
*RUNX1*	21q22.12	RUNX family transcription factor 1	Nucleoplasm; Vesicles	Low	Low	Activator; DNA-binding; Repressor	Mice	[20,43]
*SPI1*	11p11.2	Spi-1 proto- oncogene	Nucleoplasm	Bone marrow; Lung; Lymphoid tissue	Low	Activator; DNA-binding; RNA-binding	Mice	[8,44]
*TGFB1*	19q13.2	Transforming growth factor beta 1	Golgi apparatus; Cytosol	Low	Low	Growth factor; Mitogen	Mice	[20]
*TLN1*	9p13.3	Talin 1	Focal adhesion sites; Cytosol; Plasma membrane; Centriolar satellite	Low	Low	Cell-cell contact	Mice	[40]

Data are retrieved from “The Human Protein Atlas” [57] and “Gene” database of the National Center for Biotechnology Information [58]. Ref.: references; BAMs: border-associated macrophages.

## 4. BAMs vs. Microglia

### 4.1. Differences in Morphology and Motility

The BAM subpopulations and microglia generally vary in morphology. Meningeal macrophages are flat, spindle-shaped cells with a few thick membrane projections from their cell body residing close to meningeal vessels [59,60]. Although sdΜΦ appeared elongated with a more amoeboid morphology than microglia, dmΜΦ was also suggested to be pleomorphic and dendriform-like cells [8,61]. In the same context, the pvΜΦ had elongated cell bodies permitting the embedding within vessel walls [8,59]. Regarding the choroid plexus, cpΜΦ display a star-like shape, while cp^epi^ΜΦ seem more phenotypically diverse, varying from round to bipolar to stellate [8,61,62]. Under homeostatic conditions, microglia are ramified [8]. Inflammatory stimuli could shift microglial morphology from ramified to amoeboid by enlarging the cell body and retracting processes [24].

The BAMs and microglia appeared to slightly differ in motility as well. During homeostasis, pvΜΦ were non-motile, with only cellular processes extending through the blood vessel wall, whereas dmΜΦ and sdΜΦ exhibited limited motility in in vivo imaging [8,59]. Microglial cells are characterized by highly dynamic projections and a cell body with limited motility [8]. During inflammation, pvΜΦ extend dendritic-like processes along the perivascular space, indicating a potential chemotactic activation from surrounding cells, while meningeal macrophages prolongate their existing protrusions [63,64]. The amoeboid microglia exhibit a high phagocytic and proinflammatory phenotype [24]. As the in vivo imaging of choroid plexus remains challenging due to their deep localization in the ventricular system, the motility of cpΜΦ and cp^epi^ΜΦ is yet unexplored [59].

### 4.2. Differences in Biological Role

Although microglia and BAMs are immune-competent cells of the CNS with common progenitors, their different localization may contribute to variations in their biological roles. The microglial populations’ functions have been reviewed in detail [65,66,67]. Concisely, microglial cells are involved in developmental processes, including cell positioning, survival, myelinogenesis, synaptic patterning, and axonal dynamics [68]. In adult CNS, microglia, as the regulators of acute and chronic immune responses, are implicated in removing pathogens and noxious particles, scavenging cellular debris and synapses, protecting neural tissue, and mediating neurogenesis in CNS injury [24,66,69].

The unique localization of BAMs between brain parenchyma and peripheral tissues pinpoint their pivotal role in the immune surveillance of pathological antigens [16]. Their antigen-presenting capacity is attributed to MHC II molecules on some BAM surfaces [12,32,70,71]. Furthermore, the pvΜΦ and dmΜΦ mainly phagocytose intruding pathogens and any foreign molecule or substance that can be detected in the bloodstream and cerebrospinal fluid [72,72]. The pvΜΦ also appear to regulate the accessibility of brain parenchyma to circulating cells and molecules by increasing the contractility of regional vessels and capillaries or diminishing the BBB permeability [73,74,75,76]. Interestingly, the latest approaches demonstrate the involvement of BAMs in ensuring a well-balanced metabolic environment for neurons, especially in the course of systemic perturbations [77,78].

### 4.3. Differences in Molecular and Genetic Profile

The distinction between parenchymal and non-parenchymal tissue-resident macrophages, namely, microglia and BAMs, respectively, remains challenging. Identifying specific surface protein expression patterns and the genetic signature of each cell’s population could assist in studying microglia and BAMs. Both microglia and BAMs share some common surface markers. Specifically, typical macrophage markers such as C-X3-C motif chemokine receptor 1 (CX3CR1), adhesion G protein-coupled receptor E1 (ADGRE1 or F4/80), Mer tyrosine kinase (MERTK), and CD11b are present in both cell populations [5,8]. Furthermore, CSF1R, allograft inflammatory factor 1 (AIF1 or IBA1) and protein tyrosine phosphatase receptor type C (PTPRC or CD45) constitute myeloid markers of these cells [8].

Although microglia and BAMs exhibit common surface markers, their discrimination may be based on their different expression levels. For instance, significantly higher levels of CD45 were observed in BAMs compared with microglial cells. However, the levels of CD45 are not a reliable marker regarding cells’ discrimination since a subset of BAMs has been found to express low levels of CD45 [5]. Microglia-specific markers are critical in identifying the parenchymal tissue-resident macrophages (Table 2). The purinergic receptor P2Y12 (P2RY12), the hexosaminidase subunit beta (HEXB), the siglech sialic acid binding Ig-like lectin H (SIGLEC-H), the transmembrane protein 119 (TMEM119), the annexin 3 (ANXA3), and the Spalt-like transcription factor 1 (SALL1) are present only in microglia and not in BAMs, allowing their unique distinction [5,8,12,36,79,80,81].

On the contrary, cell surface phenotyping has revealed distinctive BAM markers, which may be applied for their classification (Table 2). As already been pointed out, the BAMs can be localized to three main compartments: the meninges, perivascular space, and choroid plexus, with each macrophage population acquiring characteristic molecular and genetic profiles. The CD206 (or MRC1) constitutes a signature surface protein for BAMs [5,8,36]. Subsets of BAMs were distinguished through the different expression levels of major histocompatibility complex class II (MHCII), CD38, lymphatic vessel endothelial hyaluronan receptor 1 (LYVE1), and C-C motif chemokine receptor 2 (CCR2) [5]. The CD36, CD163, CD169, and LYVE1 have been detected in pvΜΦ [5,8,36,71,82,83,84]. The pvΜΦ manifested higher levels of CD45 and lower IBA1 in relation to microglia [8,83]. Only MHCII^+^ BAMs, which enriched choroid plexus and dura mater, were found to express CCR2, explaining the increased replacement and turnover rate of cpΜΦ and dmΜΦ [5]. In addition, CNS-resident macrophages may be distinguished from peripheral monocytes since the monocytes express the integrin subunit alpha 4 (ITGA4 or CD49d), integrin subunit alpha 5 (ITGA5 or CD49e), and cell division cycle 20 (CDC20) [8,85].

In terms of genetics, BAMs carry some specific genes such as *Mrc1*, platelet factor 4 (*Pf4*), membrane spanning 4-domains A7 (*Ms4a7*), stabilin 1 (*Stab1*), apolipoprotein E (*Apoe*), membrane-spanning 4-domains, subfamily A, member 6C (*Ms4a6c*), lysozyme 2 (*Lyz2*), and transforming growth factor beta induced (*Tgfbi*) [12,14]. Similarly, microglia-specific genes have been identified, forming a unique microglial transcriptional profile. These include *P2ry12, Tmem119*, secreted protein acidic and cysteine rich (*Sparc*), olfactomedin like 3 (*Olfml3*), *HexB*, *Sall1*, triggering receptor expressed on myeloid cells 2 (*Trem2*), *Siglec-H*, and solute carrier family 2 member 5 (*Slc2a5*) [5,8,12]. Although cp^epi^ΜΦ constitute a subpopulation of BAMs located in the choroid plexus, they present significant similarities in their transcriptional profile with microglia expressing *Sall1*, a signature gene for the microglial population [14].

## 5. BAMs in Neurological Diseases and Promising Therapies

The implication of BAMs in the pathogenesis of CNS diseases, especially in neurodegeneration and neuroinflammation, is a rapidly emerging field of research. Although the precise role of BAMs in diseases is not yet elucidated, recent studies have addressed their potential involvement in several pathological conditions such as Alzheimer’s disease, Parkinson’s disease, multiple sclerosis, and stroke. Further experimental studies are needed to delineate the exact pathophysiological mechanism through which methodical manipulation of BAMs can halt or even reverse the progression of the aforementioned debilitating CNS diseases.

### 5.1. BAMs in Alzheimer’s Disease

AD is a brain disorder constituting a common cause of dementia, characterized by permanent neurodegeneration in specific brain areas [86]. However, the pathophysiology of the disease is not yet fully understood. The accumulation of amyloid beta (Aβ) protein in the brain has been implicated in AD. This protein forms sticky plaques that may disrupt the interaction between brain cells, leading to inflammation and neuronal death [87]. Additionally, AD is characterized by the accumulation of the tau protein, which forms neurofibrillary tangles [88]. Patients with Alzheimer’s disease (AD) could be affected by cerebral amyloid angiopathy (CAA), which involves the pathologic deposition of Aβ within the leptomeningeal and cortical blood vessels [89]. The role of pvΜΦ in CAA progression has been investigated in a TgCRND8 mouse model of AD [90]. Hawkes and McLaurin demonstrated that the stimulation of the pvΜΦ turnover decreased cerebral CAA load. Interestingly, the clearance of CAA load was not attributed to microglia or astrocytes. These findings indicate the importance of pvΜΦ in CAA progression, suggesting that their activation could be a useful therapeutic approach for removing vascular amyloid [90].

In Tg2576 mice, the clodronate-mediated depletion of pvΜΦ reduced the production of reactive oxygen species, thereby reversing cerebrovascular dysfunction induced by Aβ. Experiments utilizing bone marrow chimeras revealed that pvΜΦ are the primary cell expressing CD36 and NOX2, which are molecular substrates for inducing cerebrovascular oxidative stress [91]. The pvΜΦ play a significant role in upregulating secreted phosphoprotein 1 (SPP1), with perivascular fibroblasts contributing to a lesser extent. SPP1 assists microglia in engulfing synapses and increases the expression of phagocytic markers such as complement C1q A chain (C1QA), granulin precursor (GRN), and cathepsin B (CTSB) in the presence of Aβ oligomers. The deletion of *Spp1* in AD mouse models prevented synaptic loss [34]. Finally, the minor replenishment of CD206^+^ BAMs and their stable turnover in a mouse AD model should be highlighted, as potential manipulations of these cells could lead to modification of AD pathology [42].

### 5.2. BAMs in Parkinson’s Disease

Parkinson’s disease (PD) is another common neurodegenerative disorder characterized by dopaminergic cell loss [92]. The accumulation of a-synuclein (α-SYN) is a distinct trait of degenerating dopaminergic neurons [93,94]. According to Guo et al., exosomes derived from microglia and CNS macrophages facilitated the transmission of α-SYN, leading to its aggregation in neurons and contributing to the development of PD [95]. Interestingly, BAMs may mediate the α-SYN related neuroinflammation by acting as antigen-presenting cells essential for initiating a CD4 T cell response [96]. The immune cell infiltration, recruitment, and antigen presentation were found to be greatly dependent on BAMs, framing their involvement in the pathogenesis of PD [96]. A JAK1/2 inhibitor, namely, AZD1480, has been considered a therapeutic option for PD by reducing α-SYN-related neuroinflammation via downregulation of the JAK/STAT pathway [97].

### 5.3. BAMs in Multiple Sclerosis

Multiple sclerosis (MS) is a debilitating neurodegenerative disease with a rising global prevalence in recent years [98]. MS features encompass neuroinflammation, demyelination, and axonal loss within the CNS [99,100]. Several mechanisms have been proposed to be implicated in the pathophysiology of MS [101,102,103]. Nevertheless, the potential role of BAMs in MS has been only recently investigated [47,104]. BAMs, as a CNS macrophage population, could potentially be involved in the MS course through the CNS-targeted autoimmunity or neurodegeneration leading to a secondary autoimmune response [105]. BAMs are presented with different phenotypes regarding their roles in each stage of the MS [106].

Particularly, Locatelli et al. identified various markers of BAMs, utilizing immunofluorescent techniques in a MS mouse model, as the neuroinflammatory lesions shifted from expansion to gradual resolution [107]. In EAE, the most widely used animal model for studying MS aspects [108,109], antigen presentation and T cell reactivation were found to be regulated by both meningeal macrophages and microglia, revealing the involvement of BAMs in the disease [110,111]. The pvΜΦ and sdΜΦ were found to be modestly increased in the EAE mouse model, with sdΜΦ population expanding during disease onset, suggesting their implication in the initial acute phase of EAE. On the contrary, the sdΜΦ population decreased during the chronic phase of the disease and pvΜΦ proliferation remained unaltered [12].

The BAMs could also exert miscellaneous functions in MS via interleukin 9 (IL9) upregulation. Donninelli et al. found that MS patients had higher IL9 levels in the cerebrospinal fluid obtained from post-mortem samples. Through flow cytometry of snap-frozen tissue blocks from the same patients’ brains, higher expression of IL9 was also observed in macrophages [112]. Additionally, the disease-mediated peroxisome injury in BAMs, leading to demyelination and axonal loss, may be prevented through treatment with 4-Phenylbutyrate, which serves as a potential therapeutic approach for halting inflammatory demyelination and the progression of MS [113]. Lastly, foamy macrophages are formed in brain regions during MS; by targeting lipophagy, remyelination can be promoted as some BAM subtypes may be involved in the aforementioned process [114,115].

### 5.4. BAMs in Other CNS Diseases

The BAMs have also been implicated in other CNS diseases, such as stroke. The study of Pedragosa et al. highlighted the major role of BAMs in different pathophysiological changes related to ischemic stroke, including the recruitment of granulocytes, increased expression of vascular endothelial growth factor (VEGF), and increased permeability of pial and cortical blood vessels [116]. The induction of ischemic stroke resulted in the proliferation and migration of CD163^+^ BAMs adopting a pro-inflammatory phenotype in the ischemic *rat* parenchyma. Although CD169^+^ perivascular macrophages were also observed to proliferate in response to ischemic stroke, they were replaced by infiltrating bone marrow-derived cells in mice. These findings were confirmed in a human model in which CD163+ cells were also accumulated in the ischemic region [36]. In the subarachnoid hemorrhage (SAH), sdΜΦ and pvΜΦ are involved in erythrocyte uptake affecting the outcome of hemorrhage. Specifically, their depletion led to the reduction of large arterioles’ inflammation and microthrombosis after SAH [117]. Ultimately, an induction of anti-inflammatory microglial/macrophage responses and subsequent neuroprotection could be achieved through the peripheral administration of interleukin 13 in cases of ischemic stroke [118].

## 6. Conclusions

Despite the fact that the origin of BAMs has been recently elucidated, the molecular drivers orchestrating their development still represent uncharted territory. Self-maintenance or even replenishment from bone marrow-derived monocytes serves as the most viable scenario behind the BAM repopulation pattern during adulthood. The precise role of BAMs in the pathogenesis of neurodegenerative and neuroinflammatory diseases should be further explored, nonetheless. Future research has to promptly focus on distinguishing between the unique properties of these cells as well as their synergistic actions and cross-reactivity with innate immune cells, especially in the context of disease. Lastly, after more BAM subtypes biomarkers have been popularized, lab-approached manipulations could target these specific populations according to disease course and progression.

## Figures and Tables

**Table 2:** Morphology, motility, and specific surface markers of microglia and BAMs.

Cell Type	Morphology	Motility	Cell-Specific Markers
Microglia	Ramified in homeostasis; Amoeboid in inflammation	Cell bodies with limited-motility but highly dynamic processes in homeostasis; Highly phagocytic in inflammation	SIGLEC-H^+^, P2RY12^+^, HEXB^+^, TMEM119^+^, ANXA3^+^, SALL1^+^
pvΜΦ	Slightly elongated cell bodies	Non-motile cell bodies with extending and retracting projections through the blood vessel wall in homeostasis; Dendritic-like processes in inflammation	CD206^+^, CD38^+^, LYVE1^+^, CD36^+^, CD163^+^, CD169^+^
dmΜΦ	Elongated; Spindle-shaped cells; Few thick membrane projections; Dendriform	Limited motility and highly dynamic protrusions in homeostasis; Extending projections in inflammation	
sdΜΦ	Elongated; Amoeboid; Spindle-shaped cells; Few thick membrane projections	Limited motility and highly dynamic protrusions in homeostasis; Extending projections in inflammation	
cpΜΦ	Star-like shape	Unknown	
cp^epi^ΜΦ	Round; Bipolar; Stellate	Unknown	

BAMs: border-associated macrophages; pvΜΦ: perivascular macrophages; dmΜΦ: dural macrophages; sdΜΦ: subdural macrophages; cpΜΦ: stromal choroid plexus macrophages; cp^epi^ΜΦ: choroid epiplexus macrophages; SIGLEC-H: siglech sialic acid binding Ig-like lectin H; P2RY12: purinergic receptor P2Y12; HEXB: hexosaminidase subunit beta; TMEM119: transmembrane protein 119; ANXA3: annexin 3; SALL1: Spalt like transcription factor 1; CD206: Cluster of differentiation molecule 206; CD38: Cluster of differentiation molecule 38; CD36: Cluster of differentiation molecule 36; CD163: Cluster of differentiation molecule 163; CD169: Cluster of differentiation molecule 169; LYVE1: Lymphatic vessel endothelial hyaluronan receptor 1.

## Data Availability

Not applicable.

## Abbreviations

α-SYN	a-synuclein
AD	Alzheimer disease
ADGRE1	Adhesion G protein-coupled receptor E1
AIF1	Allograft inflammatory factor 1
ANXA3	Annexin 3
APOE	Apolipoprotein E
Aβ	Amyloid beta
BAMs	Border-associated macrophages
BATF3	Basic leucine zipper transcriptional factor ATF-like 3
BBB	Blood–brain barrier
c-MAF	MAF bZIP transcription factor
C1QA	C1q A chain
CAA	Cerebral amyloid angiopathy
CAMs	CNS-associated macrophages
CCR2	C-C Motif chemokine receptor type 2
CD163	Cluster of differentiation molecule 163
CD169	Cluster of differentiation molecule 68
CD206	Cluster of differentiation molecule 206
CD36	Cluster of differentiation molecule 36
CDC20	Cell division cycle 20
CNS	Central nervous system
cp^epi^ΜΦ	Choroid epiplexus macrophages
cpΜΦ	Stromal choroid plexus macrophages
CSF	Cerebrospinal fluid
CSF1	Colony stimulating factor 1
CSF1R	Colony stimulating factor 1 receptor
CTSB	Cathepsin B
CX3CR1	C-X3-C motif chemokine receptor 1
dmΜΦ	Dural macrophages
E	Embryonic day
EAE	Experimental autoimmune encephalomyelitis
EMPs	Erythro-myeloid progenitors
FIRE	Fms-intronic regulatory element
GRN	Granulin precursor
HEXB	Hexosaminidase subunit beta
IGF1R	Insulin-like growth factor 1 receptor
IL9	Interleukin 9
IRF8	Interferon regulatory factor 8
ITGA4	Integrin subunit alpha 4
ITGA5	Integrin subunit alpha 5
ITGB1	Integrin subunit Beta 1
JAK1/2	Janus kinase 1/2
LYVE1	Lymphatic vessel endothelial hyaluronan receptor 1
LYZ2	Lysozyme 2
MAFB	MAF bZIP transcription factor B
MERTK	Mer tyrosine kinase
MHCII	Major histocompatibility complex class II
MRC	Mannose receptor C-type 1
MS	Multiple sclerosis
MS4A6C	Membrane-spanning 4-domains, subfamily A, member 6C
MS4A7	Membrane spanning 4-domains A7
MYB	MYB proto-oncogene, transcription factor
NR4A1	Nuclear receptor subfamily 4 group A member 1
OLFML3	Olfactomedin like 3
P2RY12	Purinergic receptor P2Y12
PD	Parkinson’s disease
PF4	Platelet factor 4
PTPRC	Protein tyrosine phosphatase receptor type C
pvΜΦ	Perivascular macrophages
RUNX1	Runt-related transcription factor 1
SAH	Subarachnoid hemorrhage
SALL1	Spalt like transcription factor 1
sdΜΦ	Subdural/leptomeningeal macrophages
SIGLEC-H	Siglech sialic acid binding Ig-like lectin H
SLC2A5	Solute carrier family 2 member 5
SPARC	Secreted protein acidic and cysteine rich
SPP1	Secreted phosphoprotein 1
STAB1	Stabilin-1
TGF-β	Transforming growth factor beta
TGF-βR	Transforming growth factor beta receptor
TGFBI	Transforming growth factor beta induced
TLN1	Talin 1
TMEM119	Transmembrane protein 119
TREM2	Triggering receptor expressed on myeloid cells 2
VEGF	Vascular endothelial growth factor
VSMCs	Vascular smooth muscle cells
